# Dual antiplatelet therapy reduced stroke risk in transient ischemic attack with positive diffusion weighted imaging

**DOI:** 10.1038/s41598-020-75666-6

**Published:** 2020-11-05

**Authors:** Lu-lu Pei, Pei Chen, Hui Fang, Yuan Cao, Yi-nan Guo, Rui Zhang, Lu Zhao, Yuan Gao, Jun Wu, Shi-lei Sun, Xiao-ying Wang, Eng H. Lo, Ferdinando S. Buonanno, Ming-ming Ning, Yu-ming Xu, Bo Song

**Affiliations:** 1 Department of Neurology, Henan Key Laboratory of Cerebrovascular Disease, the First Affiliated Hospital of Zhengzhou University At Zhengzhou, 1st Jianshe Eastern Road, Zhengzhou, 450052 Henan China; 2 Departments of Radiology and Neurology, Massachusetts General Hospital and Harvard Medical School, Boston, MA USA; 3 Department of Neurology, Clinical Proteomics Research Center and Cardio-Neurology Clinic, Massachusetts General Hospital, Harvard Medical School, Boston, MA USA

**Keywords:** Diseases, Medical research, Neurology

## Abstract

Dual antiplatelet therapy (DAPT) reduced stroke risk in high-risk transient ischemic attack (TIA) patients assessed by ABCD2 score. Patients with positive diffusion-weighted imaging (DWI) were identified as imaging-based high-risk. The present study aims to investigate whether DAPT could reduce stroke risk in TIA with DWI positive. The study enrolled TIA patients within 72 h of onset from the prospective TIA database of the First Affiliated Hospital of Zhengzhou University. The predictive outcome was ischemic stroke at 90-day. The relationship between DAPT and stroke was analyzed in a cox proportional hazards model. The Kaplan–Meier curves of TIA patients with DAPT and monotherapy were plotted. Total of 661 TIA patients were enrolled, 279 of whom were DWI positive and 281 used DAPT. The 90-day stroke risk was higher in patients used monotherapy than those used DAPT in TIA with positive DWI (23.7% vs. 13.4%, *p* = 0.029). DAPT was associated with reduced stroke risk in TIA patients with positive DWI (hazard ratio [HR] = 0.54; 95% confidence interval [CI], 0.30–0.97; *p* = 0.037). However, the benefit didn’t exist in TIA patients with negative DWI (HR = 0.43; 95% CI, 0.14–1.33; *p* = 0.142). Early use of DAPT reduced stroke risk in TIA patients with positive DWI.

## Introduction

Several studies showed that high risk transient ischemic attack (TIA) patients could benefit from dual antiplatelet therapy (DAPT: clopidogrel plus aspirin), in short term^1,2^. However, a recent study^3^ showed that 21.8% of all patients who receive standard antiplatelet therapy experience early neurological deterioration and 9.4% suffer a recurrent ischemic stroke due to antiplatelet agent resistance. The Fast Assessment of Stroke and Transient Ischemic Attack to Prevent Early Recurrence (FASTER) trial^4^ demonstrated a trend toward a decreased rate of ischemic stroke in TIA or minor stroke patients with DAPT. Both the Clopidogrel in High-Risk Patients with Acute Non-Disabling Cerebrovascular Events (CHANCE) trial^2^ and the Platelet-Oriented Inhibition in New TIA and Minor Ischemic Stroke (POINT) trial^1,5^ showed that DAPT could decrease stroke risk in high-risk TIA patients (ABCD2 score ≥ 4) and minor stroke patients.

A recent study^6^ showed that neuroimaging parameters, but not clinical scores, were associated with recurrent cerebrovascular events among patients with minor stroke or TIA. While not all TIA patients with positive diffusion-weighted imaging (DWI) have ABCD2 score ≥ 4, some patients with ABCD2 score < 4 also suffered a high risk of stroke. Therefore, the efficacy of DAPT in TIA patients with acute positive DWI is worth being explored. This study aimed to identify whether DAPT could decrease early stroke risk in TIA patients with acute positive DWI in real-world experience.

## Methods

### Patients enrollment

The study consecutively enrolled hospitalized patients from the TIA database of the First Affiliated Hospital of Zhengzhou University. The background and methods of the database were described in other articles^7–10^. In brief, the database study was a prospective, consecutive, ongoing hospital-based registry enrolled TIA patients within 7 days of onset inaugurated in October 2010. TIA was diagnosed based on World Health Organization (WHO) diagnostic criteria, which define a TIA as an acute loss of focal cerebral or ocular dysfunction lasting less than 24 h attributed to embolic or thrombotic vascular diseases^11^. The index TIA was defined as the latest attack of the symptom leading to the most recent assessment by a neurology specialist.

All non-cardio-embolism patients were enrolled within 72 h of index TIA onset. The key exclusion criteria were: refusing to participate in the study; known severe disorder (i.e. recent cancer and hepatic disease) with a shortened life expectancy; receiving endovascular therapy or having undergone surgery for Moyamoya disease; unavailable DWI information; and patients who did not take any antiplatelet medicines during hospitalization.

All detailed baseline data of enrolled TIA patients, including demographics, clinical features, imaging features, ABCD^2^ score and antiplatelet agent, were recorded by trained physicians using paper case report forms (CRFs).

### Ethical statement

The ethics committee of the First Affiliated Hospital of Zhengzhou University approved the study and informed consents were obtained from participants. All methods were carried out in accordance with relevant guidelines and regulations.

### Definitions

In the present observational study, DAPT during hospitalization was defined as clopidogrel at a dose of 75–300 mg per day plus aspirin at a dose of 100–300 mg per day before the recurrence of stroke. Monotherapy was defined as clopidogrel (75–300 mg) or aspirin (100–300 mg) per day.

Antihypertensive and hypoglycemic therapy referred to taking any kind of relevant medications before the recurrence of stroke. Information on demographics and cardiovascular risk factors, including history of hypertension (HTN), diabetes mellitus (DM), coronary heart disease (CHD), and atrial fibrillation (AF), were obtained from patients’ self-report, medical records or treatment data. To exclude cardiogenic TIA, all patients received electrocardiogram examination or transthoracic and transesophageal echocardiography. Large-artery atherosclerosis (LAA) stroke was derived from the Trial of Org 10,172 in Acute Stroke Treatment (TOAST) stroke subtype classification system^12^. Majority of TIA patients were presented with DWI negative.Thus, the definition of LAA in the present study was modified as follows: ① patients with stenosis or (> 50%) stenosis or occlusion of a major brain artery (either intracranial or extracranial) presumably due to atherosclerosis, ② clinical findings include those of cerebral cortical impairment or brain stem or cerebellar dysfunction, ③ exclude potential sources of cardiogenic embolism. The brain imaging was not required for the diagnosis of LAA in our research. Artery stenosis was assessed by the North American Symptomatic Carotid Endarterectomy Trial (NASCET) method^13^, which defined as ≥ 50% narrowing in the lumen of the ipsilateral carotid artery by carotid artery ultrasonography, magnetic resonance angiography, computed tomographic angiography or digital subtraction angiography. DWI positive was defined as hyperintensity lesions on the b = 1,000 image with corresponding hypointensity on the apparent diffusion coefficient (ADC) maps. Two experienced neuroimaging physicians evaluated the imaging information. Inter-rater reliability of imaging assessment between two examiners blinded to patients’ information was 0.97.

### Follow-up

The predictive outcome was ischemic stroke by the 90-day follow-up. All registered patients were followed up by a neurologist blinded to the clinical information. All patients who were suspected to have a stroke were followed up through a face-to-face interview. Stroke was defined as the sudden onset of neurological symptoms persisting for ≥ 24 h based on WHO criteria^11^.

### Statistical analysis

Statistical analysis was performed with SPSS (version 19.0). A Chi-square test was used to examine the association of the baseline characteristics or risk factors of interest for categorical variables, as appropriate. A Student’s t-test was performed to determine whether there was a significant difference between groups for continuous risk factors. Kappa test was used to determine whether the ABCD^2^ ≥ 4 and DWI positivity were significantly different risk stratification methods. The risk factors associated with 90-day stroke were analyzed by Cox proportional hazards regression model. All factors of interest were included in a multivariable model. Associations were presented as hazard ratio (HR) with corresponding 95% confidence interval (CI). *p* < 0.05 (two-sided) was considered statistically significant.

## Results

**Figure 1:**
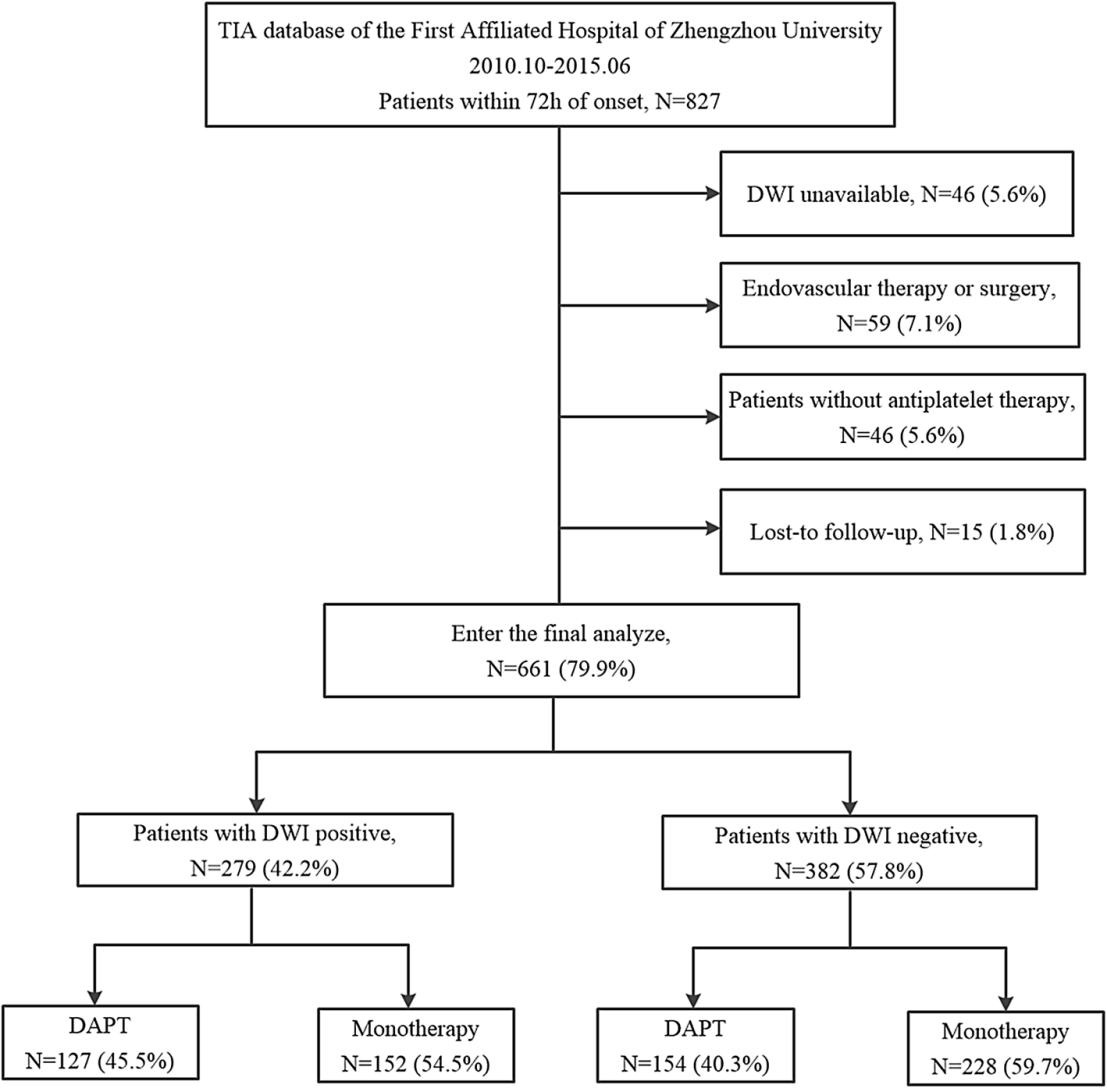
Flow diagram of the eligible TIA patients. A total of 827TIA patients within 72 h of onset were prospectively enrolled from October 2010 to June 2015. Excluded were 46 with unavailable DWI information, 59 with endovascular therapy or surgery, 46 without antiplatelet therapy, 15 patients lost-to follow-up at 90 days. In patients with DWI positive, 127 patients (45.5%) used DAPT therapy. And in patients with DWI negative, 154(40.3%) patients used DAPT therapy.

From October 2010 to June 2015, a number of 827 (86.7%) TIA patients within 72 h ictus were enrolled from the TIA database. A total of 166 (20.1%) patients were excluded according to the exclusion criteria. Figure 1 showed the detailed patients’ flow diagram. Ultimately, 661 patients were analyzed in the study, 279 (42.2%) of which were diagnosed with a positive DWI. The characteristics of patients included in the analysis were similar to those excluded (Table 1).

**Table 1:** Comparison of the clinical characteristics of the data included and excluded.

Characteristic	Analyzed (n = 661)	Excluded (n = 166)	*p* Value
Age (mean ± SD)	57.35 ± 12.43	55.58 ± 14.56	0.114
Female, n(%)	254 (38.4%)	62 (37.3%)	0.798
**Medical history, n(%)**			
Stroke	134 (20.3%)	34 (20.5%)	0.952
CHD	72 (10.9%)	25 (15.1%)	0.136
DM	108 (16.3%)	25 (15.1%)	0.688
HTN	369 (55.8%)	88 (53.0%)	0.515
Dyslipidemia	126 (19.1%)	31 (18.7%)	0.909
Current smoker, n(%)	172 (26.0%)	33 (19.9%)	0.101
ABCD2 score ≥ 4, n(%)	279 (42.2%)	75 (45.2%)	0.489

CHD, coronary heart disease; DM, diabetes mellitus; HTN, hypertension.

### Baseline information

Among the TIA patients, the average age was 57.35 ± 12.43 years, 254 patients (38.4%) were female and 281 patients (42.5%) received DAPT. Percentages of 45.5% (N = 127 /279) and 40.3% (N = 154 /382) were received DAPT in patients with positive and negative DWI, respectively. Table 2 showed the comparison of the baseline characteristics of patients with positive and negative DWI.

**Table 2:** Baseline, treatment agents during hospitalization and outcome of TIA patients with positive and negative DWI.

	DWI positive (N = 279)	DWI negative (N = 382)	*p* Value
Age	56.94 ± 12.93	57.65 ± 12.05	0.472
Female	104 (37.3%)	150 (39.3%)	0.603
HTN	163 (58.4%)	206 (53.9%)	0.250
Hyperlipidemia	53 (19.0%)	73 (19.1%)	0.971
DM	45 (16.1%)	63 (16.5%)	0.901
Previous stroke	60 (21.5%)	74 (19.4%)	0.500
CHD	22 (7.9%)	50 (13.1%)	0.034
Current smoker	75 (26.9%)	97 (25.4%)	0.667
ABCD2 score ≥ 4	138 (49.5%)	141 (36.9%)	0.001
LAA	125 (44.8%)	101 (26.4%)	< 0.001
**Treatment agents during hospitalization**			
Statins therapy	258 (92.5%)	353 (92.4%)	0.975
Antihypertension	103 (38.6%)	126 (34.0%)	0.231
Hypoglycemic agent	55 (20.6%)	58 (15.6%)	0.105
DAPT	127 (45.5%)	154 (40.3%)	0.181
90-day stroke	53 (19.0%)	17 (4.5%)	< 0.001

DAPT, dual antiplatelet therapy; DWI, diffusion-weighted image; HTN, hypertension; DM, diabetes mellitus; CHD, coronary heart disease; LAA, large-artery atherosclerosis; *p* value shows the differences between DWI positive and negative TIA patients.

Among the TIA patients with DWI positive, there was a higher prevalence of ABCD2 score ≥ 4 (*p* = 0.001) and LAA (*p* < 0.001), while also exhibiting a lower prevalence of CHD (*p* = 0.034). The number of patients received DAPT in the two groups had no statistical significance (*p* = 0.181). The other risk factors and treatment agents had no difference between the two groups.

### The Kappa test between patients assessed by two risk stratification methods

The kappa value was 0.126 (*p* = 0.001), which was less than 0.4. This result shows that the consistency of the two risk stratification methods is poor. In the other words, the patients with ABCD2 ≥ 4 and the patients with DWI positive were different.

### 90-day outcomes

In all patients, 70 patients (10.6%) experienced a stroke at the 90-day follow-up. The 90-day stroke risk was higher in patients with DWI positive compared with negative patients (18.9% VS 4.5%, *p* < 0.001, Table 2) Among DWI positive patients, 90-day stroke risk was 13.4% (N = 17) with DAPT, compared with 23.7% (N = 36) with monotherapy (*p* = 0.027). In DWI negative TIA patients, the stroke risk had no statistical significance between patients with DAPT and monotherapy (2.6% VS 5.7%, *p* = 0.149). No hemorrhagic stroke or fatal hemorrhage was observed in the study.

### Prediction factors of recurrence stroke at 90d follow-up

In TIA patients with DWI positive, the univariate Cox regression analyses showed that DAPT (HR, 0.53; 95% CI, 0.30–0.94; *p* = 0.029) was associated with reduced stroke risk at a 90-day follow-up. However, no benefit was found in DWI negative group (HR, 0.45; 95% CI, 0.15–1.37; *p* = 0.158). After adjusting for sex, CHD, LAA, ABCD2 score ≥ 4, the multivariate Cox regression analysis showed that DAPT independently reduced 90-day stroke risk (adjust HR, 0.54; 95% CI, 0.30–0.97; *p* = 0.037) in TIA patients with DWI positive. The same result could not be obtained in TIA patients with DWI negative (adjust HR, 0.43; 95% CI, 0.14–1.33; *p* = 0.142). (Table 3).

**Table 3:** Multivariate cox regression analysis for 90-days ischemic stroke occurrence.

	DWI positive		DWI negative	
	HR (95%CI)	*p* value	HR (95%CI)	*p* value
Female	1.48 (0.86–2.55)	.158	0.84 (0.31–2.31)	.737
CHD	0.22 (0.03–1.57)	.130	0.36 (0.05–2.82)	.333
LAA	1.25 (0.73–2.15)	.415	0.68 (0.19–2.39)	.545
ABCD2 ≥ 4	1.49 (0.86–2.58)	.154	2.15 (0.83–5.62)	.117
DAPT	0.54 (0.30–0.97)	.037	0.43 (0.14–1.33)	.142

DWI, diffusion-weighted image; CHD, coronary heart disease; LAA, large-artery atherosclerosis; DAPT, dual antiplatelet therapy.

**Figure 2:**
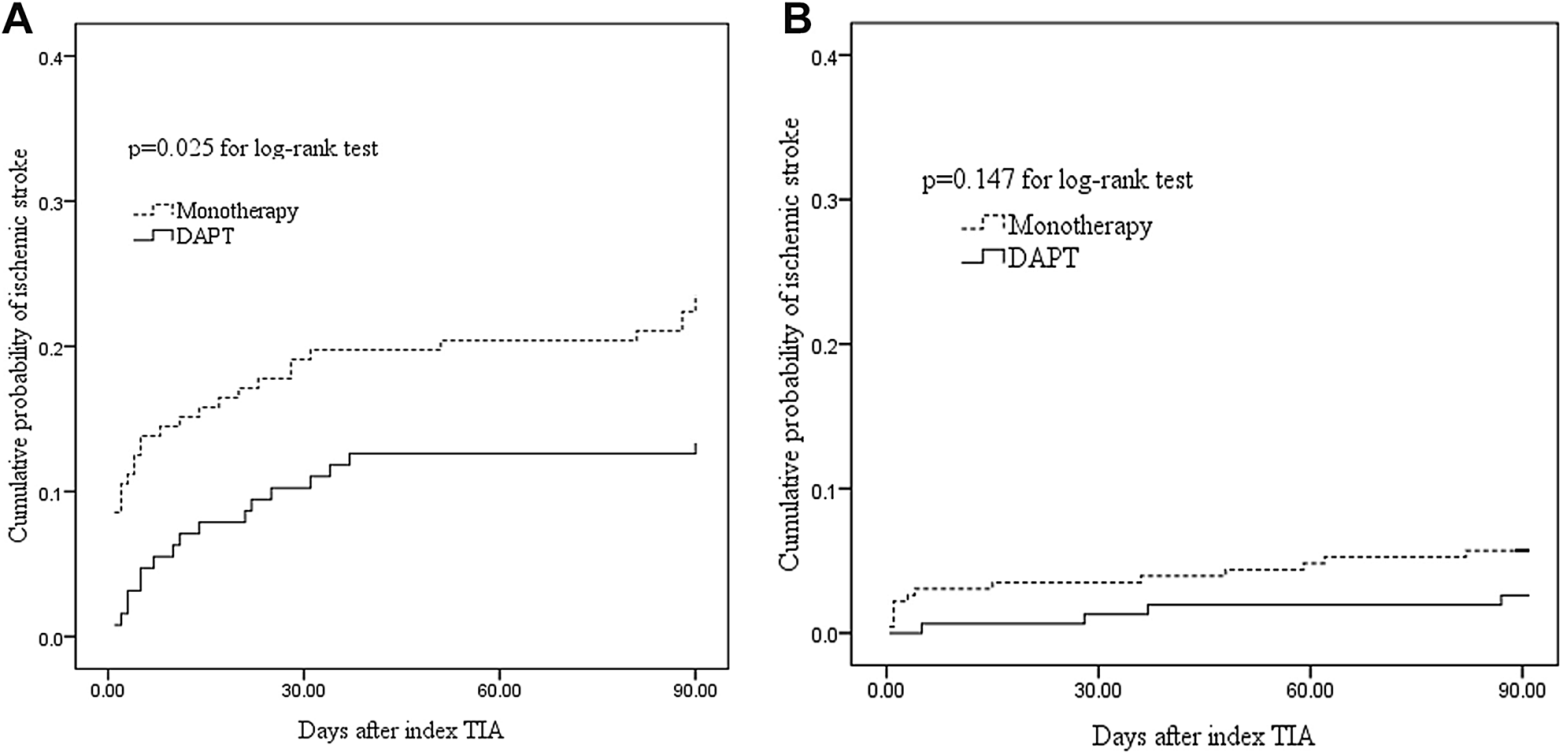
Cumulative probability of ischemic stroke in TIA patients with monotherapy and DAPT by DWI patterns. In TIA patients with DWI positive, there was a significant difference in the 90-day stroke rate between DAPT and monotherapy (log-rank test = 4.994, *p* = 0.025, **A**). However, the difference was not statistical significance in patients with DWI negative (log-rank test = 2.106, *p* = 0.147, **B**).

The survival-free-of-stroke curves of DAPT and monotherapy for different groups of TIA patients were shown in Fig. 2. There was a significant difference in the 90-day stroke rate between DAPT and monotherapy in TIA patients with DWI positive (log-rank test = 4.994, *p* = 0.025), but not in TIA patients with DWI negative (log-rank test = 2.106, *p* = 0.147).

## Discussion

Our study advanced present knowledge by incorporating DWI imaging selection of TIA patient which is a different cohort from those assessed by clinical scales. The study showed that early-use of aspirin plus clopidogrel is associated with a 46% decrease of 90-day stroke risk in high-risk TIA patients with DWI positive.

In the present study, we found that the risk of stroke is much higher in TIA patients with positive DWI, sharing a similar result with other studies published^14–16^. So far, it is still not clear which population could gain more benefits from DAPT. Some specific groups were inclined to gain benefits from DAPT in previous studies. For example, in the FASTER trial, TIA patients with more serious clinical symptoms which lasted for greater than five minutes, such as speech or weakness disturbance, dysphasia or dysarthria, were eligible. Patients with an ABCD2 score ≥ 4 or acute minor stroke (NIHSS score ≤ 3) were enrolled in the CHANCE and POINT study. However, tissue-based high-risk TIA patients with positive DWI were not studied in previous study, which might be a potential group. In this study, these patients could also benefit from DAPT.

Although the ABCD2 score has been widely used to triage TIA patients who are at high risk for impending stroke, its predictive value has been questioned^7,17,18^. Many studies have suggested that TIA patients with DWI positive have a higher stroke risk than DWI negative ones^14^. A previous study indicated that TIA patients with DWI positive were more likely to have unilateral weakness, a TIA lasting more than 60 min, ABCD2 > 5, LAA and AF^19^. Thus, DWI might be a new predictor for those patients who are suitable to receive DAPT. In this study, there were 279 TIA patients (42.2%) with DWI positivity. There were 138 TIA patients (49.5%) with an ABCD2 score ≥ 4 among TIA patients with DWI positive. The cut-off point of TIA patients with DWI positive was 4.5. Moreover, the Kappa Test showed that the patients with ABCD^2^ ≥ 4 and the patients with DWI positive were different. In this study, TIA patients with positive DWI had a 46% decrease risk of stroke after DAPT. The finding of the study had important clinical significance. In the clinical practice, clinicians should give an overall evaluation including both clinical predictive scores and imaging information before DAPT is administered to TIA patients. However, the evidence for DAPT benefits needs further randomized controlled trials to support, especially for those with non-disabling ischemic cerebrovascular events with tissue-based definition.

No major adverse events, such as intracranial hemorrhaging or gastrointestinal hemorrhaging, occurred during this study. The result was consistent with a lower rate of hemorrhage in CHANCE trial. In our study, only 5.6% of patients received DAPT at 90-day follow up, which means about 95% TIA patients received short-term DAPT. A meta-analysis^20^ including 14 studies of 9012 patients showed that when non-cardioembolic ischemic stroke or TIA with treatment initiated within 3 days of ictus, short-term DAPT significantly reduced the risk of stroke recurrence (risk ratio, 0.69; 95%CI, 0.60–0.80; *p* < 0.001), compared with monotherapy, and non-significantly increased risk of major bleeding (risk ratio, 1.35; 95% CI, 0.70–2.59; *p* = 0.37). In this study, the patients who suffered TIA in last 3 days before hospitalization were enrolled and these patients received short-term DAPT.

Since it is an observational study, we cannot design the length of DAPT treatment time. There were some limitations in our study. First, we simplified the definition of the DAPT management because of the complex and flexible individualized treatment in real clinical conditions which might result in some bias. Second, the duration of DAPT use after hospital discharge may affect the results. Third, patients with much more risk factors for recurrence stroke were more likely to be treated with DAPT. But these patients were from a prospective cohort and the confounding stroke risk factors were adjusted in the multi-variable analysis. Last, as a single center study, there may be selection bias. However, our data provide proof of concept data for the utility of DAPT in the real world.

## Summary

In conclusion, early use of DAPT could decrease short-term stroke risk in imaging-based high-risk TIA patients. Further multi-center study was needed to confirm the finding.
